# Assessment of Price and Clinical Benefit of Cancer Drugs in Canada, 2011-2020

**DOI:** 10.1001/jamanetworkopen.2022.53438

**Published:** 2023-01-31

**Authors:** Kristina Jenei, Daniel Meyers, Bishal Gyawali

**Affiliations:** 1 Department of Health Policy, London School of Economics and Political Science, London, United Kingdom; 2 Department of Medicine, University of Calgary, Calgary, Alberta, Canada; 3 Division of Cancer Care and Epidemiology, Queen’s University Cancer Research Institute, Kingston, Ontario, Canada

## Abstract

This cohort study examines the association between approval characteristics, clinical benefit, and prices of cancer drugs recommended for reimbursement by the Canadian Agency for Drug and Technology in Health.

## Introduction

Cancer drug prices have increased exponentially in the past decade and several studies have demonstrated an increasing disconnect between clinical benefit and prices for cancer drugs approved by the US Food and Drug Administration or the European Medicines Agency.^1,2^ This disconnect seemed to exist even in the setting of central price negotiations in Italy.^3^ However, to our knowledge this has not been studied for Canada. This study examined the association between approval characteristics, clinical benefit, and prices for cancer drugs recommended for reimbursement in Canada by the Canadian Agency for Drug and Technology in Health (CADTH).

## Methods

CADTH provides funding recommendations to Canadian provinces and territories (except Quebec). We conducted a retrospective cohort study of anticancer drugs for treatment of solid tumors in adult patients that received positive reimbursement recommendations from inception in 2011 to 2020. Supportive care medicines, hematologic neoplasms, pediatric indications, and biosimilars were excluded, which allowed for a homogenous cohort to evaluate clinical benefit using the ESMO-MCBS (European Society for Medical Oncology-Magnitude of Clinical Benefit Scale).^4^ In this scale, a score of 4 or 5 in the metastatic setting or A or B in adjuvant settings were considered substantial clinical benefit. We extracted supporting trials characteristics and monthly drug prices from CADTH reports.^5^

This study examined publicly available data and did not require institutional ethics approval as per the research ethics policy and procedures at the London School of Economics and Political Science. This study followed the Strengthening the Reporting of Observational Studies in Epidemiology (STROBE) reporting guideline.

While prices do not reflect confidential discounts, estimates are based on data submitted by the manufacturer and reanalyzed by CADTH. Kruskal-Wallis and Mann-Whitney tests were used to determine the association between approval characteristics and prices. Linear regression was used to examine the association between percentage improvement in progression-free survival (PFS) and overall survival (OS) with monthly prices using R Statistical Software version 3.5.0 (R Project for Statistical Computing). *P* < .05 in 2-sided tests was considered statistically significant. All prices are reported in US dollars.

## Results

Between 2011 and 2020, there were 78 positive reimbursement recommendations for solid tumors. Of these, 30 (38%) were for novel drugs, 41 (53%) for new indications of existing drugs, and 7 (9%) were resubmissions that received previous negative decisions. All submissions had evidence from a phase 2 or phase 3 clinical trial.

Drugs that offered substantial clinical benefit were associated with a higher median monthly price ($6207; range, $1723-$34 305) compared with low benefit ($4437; range, $782-$11 733) per ESMO-MCBS (*P* < .001) (Table). Immune checkpoint inhibitors were priced highest ($8533; range, $5668-$34 305). There was a significant difference between the median monthly treatment costs of drugs that received recommendations for melanoma compared with other tumor types (eg, monthly price: melanoma, $8342; range, $782-$34 305 vs gastrointestinal, $4293; range, $2105-$17 500; *P* < .001). Conditional recommendations were associated with higher median monthly prices compared with regular recommendations ($6184; range, $1723-$34 305 vs $3289; range, $782-$7298; *P* < .001). No other significant associations were found. We found a weak correlation between monthly treatment costs and percentage improvements in median PFS (*R^2^* = 0.040) and OS (*R^2^* = 0.361) (Figure).

**Table.** Prices and Characteristics of Cancer Medicines Recommended for Funding in Canada Between 2011 to 2020

Characteristic	Monthly price, median (range), US$^a^	*P* value^b^
**Drug**		
Total submissions	6025 (782-26 388)	NA
Type of submission		
New drug	6148 (1723-32 480)	.96
New indication	5936 (782-34 305)	
Resubmission	5683 (3188-9445)	
Type of recommendation		
Conditional	6184 (1723-34 305)	<.001
Regular	3289 (782-7298)	
Drug type		
Immune checkpoint inhibitor	8533 (5668-34 305)	<.001
Monoclonal antibody	6324 (3231-13 031)	
Small molecule inhibitor	5481 (1723-17 992)	
Hormonal therapy	2390 (2441-3497)	
Cytotoxic therapy	4526 (3515-6820)	
Other	3921 (782-17 500)	
**Trial**		
Tumor type		
Genitourinary	3289 (2441-16 302)	<.001
Melanoma	8342 (782-34 305)	
Gastrointestinal	4293 (2105-17 500)	
Breast	4706 (2690-13 031)	
Lung	6212 (1723-11 780)	
Gynecological	6184 (4200-11 615)	
Other	5647 (3231-8400)	
Treatment setting		
Adjuvant	7191 (5614-14 929)	.26
Advanced or metastatic	6025 (782-34 305)	
Single-arm		
Yes	6351 (782-24 360)	.24
No	5652 (1723-34 305)	
RCT evidence		
Yes	5812 (1723-34 305)	.75
No	6264 (782-10 933)	
Treatment line^c^		
1st	6184 (1723-34 305)	.63
2nd and beyond	6114 (782-32 480)	
Primary endpoint		
OS	6202 (2105-32 480)	.57
PFS	5376 (1723-34 305)	
RR	6203 (2442-10 933)	
Other	5521 (2442-14 929)	
Health-related quality of life		
Assessed	6184 (1723-34 305)	.17
Not assessed	4658 (782-11 615)	
ESMO-MCBS benefit		
High	6207 (1723-34 305)	<.001
Low	4437 (782-11 733)	
ESMO MCBS categories^d^		
1	NA	.03
2	4898 (2690-11 733)	
3	4164 (782-10 933)	
4	6212 (1723-34 305)	
5	6327 (6318-9035)	
A	7191 (5614-14 929)	
B	NA	

Abbreviations: ESMO-MCBS, European Society for Medical Oncology-Magnitude of Benefit Scale; NA, not available; OS, overall survival; PFS, progression-free survival; RR, response rate.

^a^ Prices reported in US$ after applying the exchange rate of September 3, 2022.

^b^ *P* values are unadjusted.

^c^ Treatment line applicable to medicines in advanced or metastatic setting.

^d^ There were no positive CADTH recommendations with an ESMO-MCBS score of 1 or B.

**Figure.**
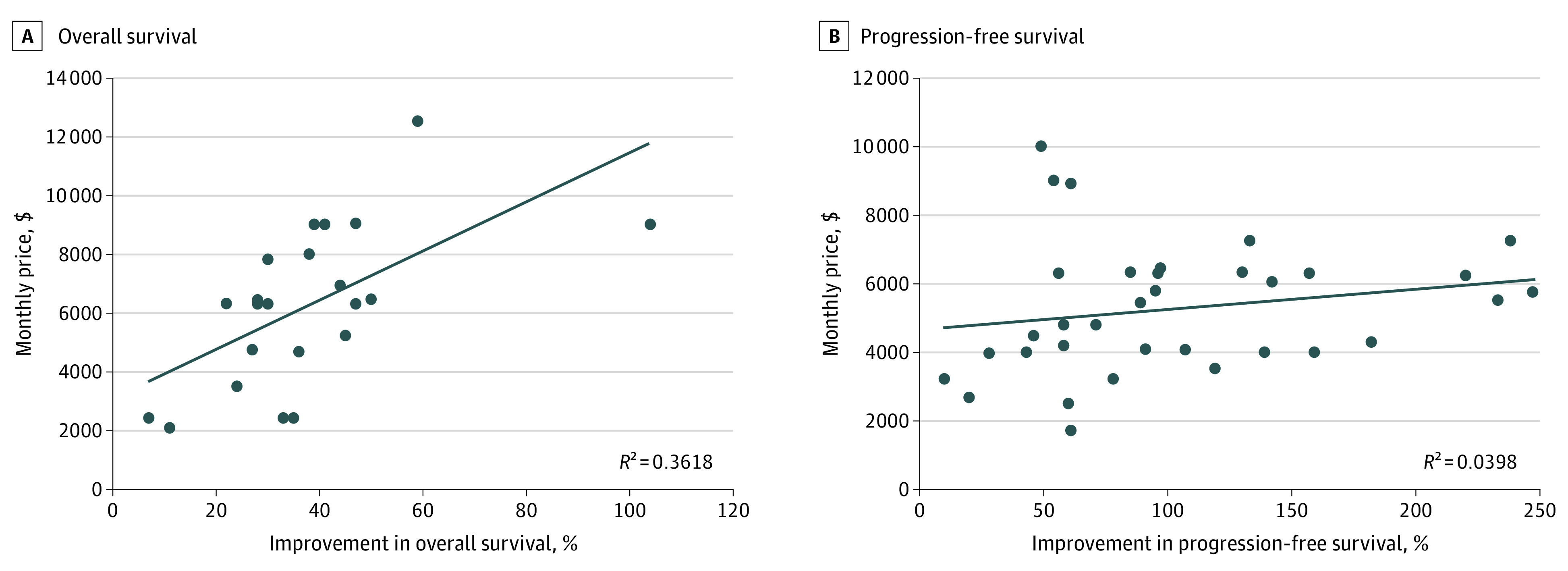
Association Between Improvement in OS and PFS and Monthly Drug Costs A total of 4 outliers were removed for consistency.

## Discussion

Unlike US or Europe, monthly prices in Canada significantly differed for cancer drugs with substantial benefit vs low benefit per MCBS scores. However, consistent with other studies,^1,2^ there was only a weak correlation between monthly drug prices and PFS or OS gains.

MCBS captures broader evidence including hazard ratios, quality of life, and toxicity. Thus, drug prices differing based on MCBS scores but not based simply on PFS or OS gains is a reassuring finding to the MCBS. However, MCBS is based solely on clinical trials, so the correlation of prices with population-level benefit remains unmeasured. These findings are important to consider for US policy makers as the legislations for price negotiation are under discussion.^6^ We have previously shown that drugs that receive a positive reimbursement recommendation in Canada, in general, have better quality of evidence and magnitude of benefit than the drugs approved by the FDA.^7^ However, having a health technology assessment should not be presumed to directly cause better alignment of drug prices since previous studies from European nations have failed to find such association despite price negotiations.^1,3^

## Notes

Supplement.
Data Sharing Statement
