# Supplementary material for: Assessment of Price and Clinical Benefit of Cancer Drugs in Canada, 2011-2020
Source: JAMA Netw Open. 2023 Jan 31;6(1):e2253438. doi: 10.1001/jamanetworkopen.2022.53438 (PMC9890281; doi:10.1001/jamanetworkopen.2022.53438)
Supplement: Supplement. — Data Sharing Statement [file jamanetwopen-e2253438-s001.pdf]

Data Sharing Statement

Jenei. Assessment of Price and Clinical Benefit of Cancer Drugs in Canada, 2011-2020. *JAMA Netw Open*. Published January 31, 2023. doi:10.1001/jamanetworkopen.2022.53438

Data
Data available: No

Additional Information
Explanation for why data not available: Data collected was publicly available data that can be downloaded from the CADTH website. In addition, researchers can contact the lead author (k.jenei@lse.ac.uk) to obtain a copy of the data, if needed.
